# A novel needle for subcutaneous injection of interferon beta-1a: effect on pain in volunteers and satisfaction in patients with multiple sclerosis

**DOI:** 10.1186/1471-2377-8-38

**Published:** 2008-10-10

**Authors:** Amer Jaber, Gian B Bozzato, Lionel Vedrine, Wes A Prais, Julie Berube, Philippe E Laurent

**Affiliations:** 1 Merck Serono International S.A., Geneva, Switzerland; 2 BD Medical – Pharmaceutical Systems, Franklin Lakes, NJ, USA; 3 BD Medical – Pharmaceutical Systems, Le Pont de Claix, France; 4 UCB Pharma SA, Chemin du Foriest, 1420 Braine l'Alleud, Belgium

## Abstract

**Background:**

To reduce injection pain and improve satisfaction, a thinner (29-gauge [29G]), sharper (5-bevel) needle than the 27G/3-bevel needle used previously to inject interferon (IFN) beta-1a, 44 or 22 mcg subcutaneously (sc) three times weekly (tiw), was developed for use in multiple sclerosis (MS).

**Methods:**

Two clinical trials in healthy volunteers and five surveys of patients with MS were conducted to assess whether the 29G/5-bevel needle with a Thermo Plastic Elastomer (TPE) needle shield (a sleeve that houses the tip of the needle in a secure location) is an improvement over the 27G/3-bevel needle with a rubber shield for injection of IFN beta-1a, 44 or 22 mcg sc tiw. Parameters assessed were: pain and ease of insertion (healthy volunteer and nurse responses on subjective pain measurement scales); and patient satisfaction (surveys of patients with MS).

**Results:**

In healthy volunteers, the 29G/5-bevel needle with TPE shield was associated with the least perceived pain on the Visual Analog Scale (VAS) and Verbal VAS (VB-VAS); mean VAS pain scores decreased by 40% and skin penetration improved by 69% compared with the 27G/3-bevel needle with standard rubber shield (p < 0.01). Pooled results from surveys of patients with MS indicated that 63% of patients thought that injections were less painful with the 29G/5-bevel needle than the 27G/3-bevel needle. Results from individual surveys indicated that the 29G/5-bevel needle was an improvement over the 27G/3-bevel needle for ease of insertion, injection-site reactions, bruising, burning and stinging.

**Conclusion:**

Together these studies indicate that the 29G/5-bevel needle with the TPE shield is an improvement over the 27G/3-bevel needle with standard rubber shield in terms of pain, ease of insertion and patient satisfaction. These improvements are expected to result in improved compliance in patients with MS treated with IFN beta-1a, 44 or 22 mcg sc tiw.

## Background

Although details of the frequency of the injections and delivery systems differ, all disease-modifying drugs (DMDs) approved for the treatment of relapsing forms of multiple sclerosis (MS) are currently administered by injection, some of which can be self-administered by the patient.

A patient's perception of the pain caused by drug delivery may directly influence convenience, compliance and acceptance of interventions with proven efficacy. Accordingly, research efforts have focused on improving the design of existing needles to reduce injection-associated pain without compromising the needle's functional integrity. For patients treated with interferon beta-1a (IFN β-1a; Rebif^®^, Merck Serono International S.A., Geneva, Switzerland [an affiliate of Merck KGaA, Darmstadt, Germany]), 44 or 22 mcg subcutaneously (sc) three times weekly (tiw), a syringe (BD Hypak Physiolis™, Becton Dickinson and Company) has been developed that is fitted with a needle that is thinner (29-gauge [29G]) and sharper (5-bevel) than the previous 27G/3-bevel needle but which maintains the same flow rate. The other unique feature of the system is the rigid needle shield made from a Thermo Plastic Elastomer (TPE) instead of rubber. The rigid needle shield is the guarantee of the quality preservation of the needle and makes needle shield removal very intuitive, thus limiting the risk of damaging the needle point.

The 'gauge' of a needle indicates its thickness – the higher the gauge, the thinner the needle. Maintenance of the flow rate with the thinner 29G/5-bevel needle is made possible by technological improvements that allow the inner diameter of the 29G/5-bevel needle to be the same as that for the 27G/3-bevel needle, although the outer diameter is reduced. Maintenance of the inner diameter also means that the same pressure is required to inject fluid once the skin has been penetrated. The '5-bevel' means that the tip is cut at five different angles, making it sharper than the previous 3-bevel design. Indeed, engineering tests using synthetic skin showed that the force required to penetrate the skin is reduced by 19–23% using the 5-bevel needle-tip design compared with a 3-bevel needle [1]. Furthermore, surveys of patients with MS support improved skin penetration and indicate that the 29G/5-bevel needle is less painful than the 27G/3-bevel needle [2] when injecting IFN β-1a, 44 or 22 mcg sc tiw.

The 29G/5-bevel needle was first made available to patients with MS in 2004 for the injection of IFN β-1a, 44 or 22 mcg sc tiw, and became available globally at the beginning of 2005. It was expected that using the 29G/5-bevel needle would reduce injection pain and make needle insertion easier, resulting in improved patient compliance. Accordingly, seven studies (two clinical trials in healthy volunteers and five surveys of patients with MS) were conducted to find out whether the 29G/5-bevel needle is, in practice, an improvement over the previous 27G/3-bevel needle in terms of pain, ease of insertion and patient satisfaction. We also assessed the reliability of three subjective pain measurement scales. We present here the results of these seven separate studies of the 29G/5-bevel needle.

## Methods

In total, seven separate studies were conducted to assess the 29G/5-bevel needle: two double-blind, randomized, clinical trials in healthy volunteers to assess injection-associated pain with the 27G and 29G needles and validate the reliability of three subjective pain measurement scales (one single-center trial in France, and a multi-center study in France and the USA); and five surveys in four countries (Australia, Denmark, Germany and the USA) to assess satisfaction with the 29G/5-bevel needle in patients who had previously administered IFN β-1a, 44 or 22 mcg sc tiw, using the 27G/3-bevel needle.

### Clinical trials

The two separate, but similar, clinical trials were designed to assess the reliability of three subjective pain scales and to establish if the 29G/5-bevel needle, currently used for the injection of IFN β-1a, 44 or 22 mcg sc tiw, is less painful and easier to insert than the previous 27G/3-bevel needle. Both trials enrolled healthy adult volunteers who had given their informed consent. These studies were conducted in accordance with the principles described in the Declaration of Helsinki, including all amendments through the 1996 South Africa revision. All sites received approval from relevant Health Authorities, from the Ethics Committee for the French site that was involved in both studies (Comité Consultatif de Protection des Personnes se Prêtant à une Recherche Biomedicale de Bourgogne, approval dated 28 November 2000 and 24 January 2002) and from the Institutional Review Board (INTEGREVIEW Inc., 1825 Fortview Road, Suite 110, Austin, Texas, approval dated 30 April 2002) for the US site of the French/US multi-center study (0148). After medical screening to confirm that volunteers were in a healthy condition, each volunteer received three needle pricks, without fluid injection, over a 3-day period from needles with different outer diameters (29G versus 27G), tip geometries (5 bevel versus 3 bevel), needle-shield material (rubber versus TPE) and lubricants. The needles used were intended to mimic the pre-filled needles used by patients with MS who self-inject. Registered nurses (12 nurses in the single-center study and 25 nurses in the multi-center study) administered needle pricks to the volunteers' abdomens using the standard sc injection technique [3]. To achieve blinding and randomization, both the nurses and the volunteers were unaware of the types of needles being used, and the volunteers' allocation to nurses and the ranking order of the various test needles were random. All needles were pre-attached on glass syringes and embedded in a rubber needle shield (except the commercially available disposable needles used for reference in the multi-center trial), in order to mimic the design of the pre-filled syringes that would be used in clinical practice.

#### Single-center French study

Four 27G and four 29G needles (all 0.5 inches in length) with different characteristics were tested (Table 1); volunteers were pricked every 10 min over two, 40-min periods/day (24 pricks in total). Immediately after each needle prick, volunteers were asked the question "How painful is it?", and their perceived pain was evaluated using the uni-dimensional Visual Analog Scale (VAS) as well as a Verbal-VAS (VB-VAS). Nurses were also asked to evaluate the ease of skin penetration using the VAS and VB-VAS, and were asked the question "How difficult is it to penetrate the skin?"

**Table 1:** Characteristics of the different needles studied in the single- and multi-center trials of healthy volunteers

**Needle gauge**	**Number of bevels**	**Needle shield material**	**Type of silicone lubricant**	**Single-center study**	**Multi-center study**
27^a^	3	Rubber	A	×	××
27	5	TPE	B	×	
27	5	Rubber	A	×	
27	5	TPE	A	×	×
27	3	Rubber	A	×	
29	3	TPE	A	×	
29	5	Rubber	A	×	
29^b^	5	TPE	A	×	×
27 disposable needle	-	-	A		×
29 disposable needle	-	-	A		×

IFN, interferon; sc, subcutaneous; tiw, three times weekly; TPE, Thermo Plastic Elastomer.

^a^Needle used previously for the injection of IFN β-1a, 44 or 22 mcg sc tiw.

^b^Needle used currently for the injection of IFN β-1a, 44 or 22 mcg sc tiw.

The primary objective of this study was to assess and characterize the impact of three needle parameters (gauge, bevel geometry and needle-shield material) on the volunteers' perception of pain and the nurses' perception of ease of needle insertion using the VAS and VB-VAS. The secondary objective was to identify the best candidate needle (*i.e. *the least painful and easiest to insert) for pre-attachment to BD Hypak Physiolis™ syringe. Another objective of the study was to establish if there is a correlation between the VAS and VB-VAS in volunteers' ratings of pain for the various needles.

The VAS is a 10-cm horizontal line labeled with pain descriptors. Volunteers indicated the magnitude of the pain associated with injection using each needle by allocating a score ranging from 0 (no pain) to 100 (very painful). The descriptors on the VAS were also modified to evaluate the nurses' perception of how much resistance was encountered when using each needle to penetrate the volunteers' epidermis. On this modified scale, nurses allocated a score ranging from 0 (no resistance) to 100 (strong resistance). This assessment was conducted for the 29G/5-bevel needle with TPE shield, the 29G/5-bevel needle with rubber shield and the 27G/3-bevel needle with rubber shield.

**Figure 1:**
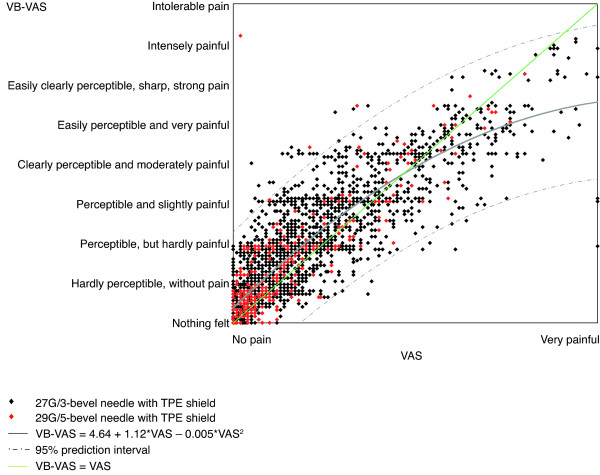
**Relationships between the Verbal Visual Analog Scale (VB-VAS) and the VAS for volunteers' perception of pain associated with injections using different needle types.** The solid grey line is the fitted line and the dashed grey lines are simultaneous 95% prediction boundaries for the whole curve. The green line represents the line of perfect agreement and the estimated equation that characterizes the relationship is: VB-VAS = 4.64 + 1.12*VAS – 0.005*VAS^2^. TPE, Thermo Plastic Elastomer.

The VB-VAS was developed from the VAS to specifically measure the pain induced by sc needle pricks [4]. It is a 10-cm vertical line labeled with nine pain intensity descriptors (see Figure 1, *y*-axis), which were adapted from the Gracely Box SL pain scale [5]. Volunteers indicate the magnitude of the pain they are experiencing by allocating each injection a score ranging from 0 (no pain) to 100 (intolerable pain). Nurses gave their assessment of needle sharpness by allocating a score ranging from 0 to 100 for each injection they administered; a score of 0 corresponded to the descriptor "impossible to insert" and a score of 100 corresponded to the descriptor "greatest ease of insertion".

#### Multi-center French/US study

**Figure 2:**
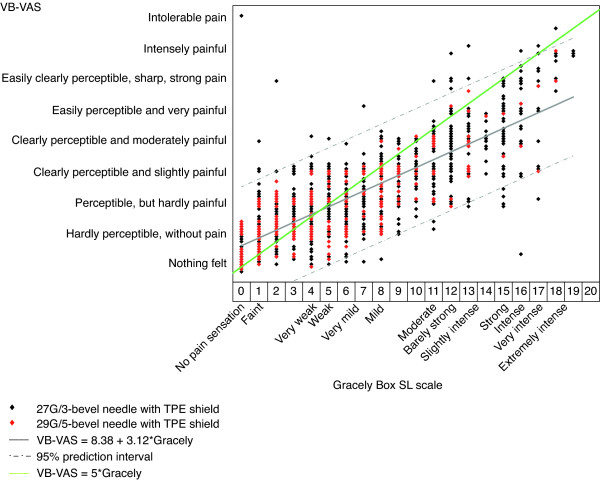
**Relationships between the Verbal Visual Analog Scale (VB-VAS) and the Gracely Box SL scale for volunteers' perception of pain associated with injections using different needle types.** The solid grey line is the fitted line and the dashed grey lines are simultaneous 95% predictions boundaries for the whole line. The green line represents the line of perfect agreement and the estimated equation that characterizes the relationship is: VB-VAS = 8.38 + 3.12*Gracely. TPE, Thermo Plastic Elastomer.

Six needles (all 0.5 inches in length) with different characteristics were tested (Table 1); healthy volunteers were pricked every 10 min over two, 30-min periods/day (18 pricks in total). Immediately after each needle prick, volunteers evaluated their perceived pain using the VB-VAS and the Descriptor Differential Scale pain scale; also referred to as the Gracely Box SL scale [5] (see Figure 2, *x*-axis). These results were then used to establish if there is a correlation between the VB-VAS and Gracely Box SL pain scale in individuals' ratings of pain for the various needles.

### Surveys

Patients with MS who were from Australia, Denmark, Germany and the USA and had been using the 27G/3-bevel needle to administer IFN β-1a, 44 or 22 mcg sc tiw, were interviewed in five separate surveys (two surveys in Australia) to establish if injections with the 29G/5-bevel needle are associated with a greater degree of satisfaction (*e.g. *reduced pain, easier to insert). In all five surveys, patients had to have been using the 29G needle for at least 2 weeks. The surveys were all conducted in countries where patients receive full reimbursement for their MS treatment. For each survey, the questions comparing the 29G/5-bevel needle with the 27G/3-bevel needle, and the multiple-choice answer options, are listed in Table 2. We report here only the questions that compared the 29G/5-bevel needle with the 27G/3-bevel needle.

**Table 2:** Questions comparing the 29-gauge [29G]/5-bevel and 27G/3-bevel needles and multiple-choice answer options for each survey (see Figures 4 and 5 for survey results)

**Australian pilot survey (n = 11)**	
How would you describe your injections with the new pre-filled syringes compared with before?	*Much less painful; Slightly less painful; Same; Quite painful; Very painful*
Does the new needle penetrate the skin more easily?	*Yes, far more easily; Yes, a bit; No, not easily at all; Don't know*
Have you noticed any difference in the needle of the pre-filled syringe?	*Yes; No*
**Main Australian survey (n = 156)**	
Has it reduced the pain associated with injection?	*Yes; No; Not indicated; Not tried*
Did you notice the difference with the thinner needle?	*Yes; No; Not indicated; Not yet tried*

**Danish survey (n = 14)**	
How painful are injections using the new pre-filled syringe?	*Much less; Slightly less; Same; Slightly more; Much more*

**German survey (n = 109)**	
How painful are the new pre-filled syringes compared with the previous injections?	*Much less painful; Slightly less painful; Same; Slightly more; Much more; No response*

**US survey (n = 78)**	
How would you compare pain with the trial syringe ...?	*No difference; Much More; Somewhat more; Somewhat less; Much less*
How would you compare ease of insertion of the trial syringe ...?	*No difference; Much more difficult; Somewhat more difficult; Somewhat easier; Much easier*
How does the trial syringe compare to the previous syringe ... is the trial syringe ...?	*No difference; Much worse; Somewhat worse; Somewhat better; Much better*
In future, which syringe would you prefer to use for your regular injections?	*Trial; Previous*
How would you compare injection-site reactions with the trial syringe?	*No difference; Much more; Somewhat more; Somewhat less; Much less*
How would you compare bruising with the trial syringe?	*No difference; Much more; Somewhat more; Somewhat less; Much less*
How would you compare stinging or burning with the trial syringe?	*No difference; Much more; Somewhat more; Somewhat less; Much less*

#### The Australian surveys (pilot and main survey)

Patients who had been using the 27G/3-bevel needle to administer IFN β-1a, 44 mcg sc tiw (with or without the Rebiject mini^® ^auto-injector [Merck Serono International S.A., Geneva, Switzerland]), for the previous 6–24 months and were registered on the Rebif^® ^Extra Care database were chosen randomly to be contacted about participation in the survey by telephone by a Clinical Educator, or by post. Patients who agreed to participate were sent pre-filled syringes fitted with 29G/5-bevel needles and responded to questions by telephone or by post after using the needle for 1 month.

#### The Danish pilot survey

Patients who had been using the 27G/3-bevel needle (with or without the auto-injector) for longer than 6 months were enrolled in the study by their treating physician. The survey questionnaire was completed at enrollment and at 4 weeks after switching to the pre-filled syringe with the 29G/5-bevel needle.

#### The German survey

Patients who had been using the 27G/3-bevel needle (with or without the auto-injector) for longer than 3 months were enrolled by their treating physician, who supplied them with the pre-filled syringes fitted with 29G/5-bevel needles. Patients completed the multiple-choice questionnaire after using the 29G/5-bevel needle for at least 1 month.

#### The US survey

A total of 150 patients who had been using the 27G/3-bevel needle for manual injection for the previous 6 months to 2 years and who were registered on the MS Life Lines database were contacted about participation in the telephone survey. Patients who agreed to participate in the telephone-based survey were sent the 29G/5-bevel needle and interviewed after using the needle for at least 2 weeks.

### Needle manufacture

All needles used in the studies were manufactured and supplied by BD. Although the 29G needles used in the studies were thinner than the previous 27G needle, the inner diameter has been preserved using thin-wall technology, allowing the same flow rate and the same pressure required to inject fluid once the skin has been penetrated.

### Study drug

For the five surveys, IFN β-1a, 44 or 22 mcg sc tiw, was supplied in pre-filled syringes. The drug formulation supplied in the pre-filled syringe was the same regardless of whether it was fitted with a 27G/3-bevel or 29G/5-bevel needle. All participants were trained to inject, by either investigation-site personnel or a trained nurse, to minimize injection-site reactions due to poor injection technique. Injection instructions included use of an alcohol swab to clean the skin at the injection site, rotation of injection sites and emphasis on the importance of avoiding already inflamed areas for subsequent injections.

### Statistical analysis

For each of the five surveys, observational statistics were employed.

#### The five surveys

The proportions of patients responding to each multiple-choice answer were calculated for each of the surveys. If common questions were employed by more than one survey, the patients' responses were pooled. However, as the wording of the multiple-choice answer options provided for common questions varied, patient responses were categorized as 'positive', 'negative' or 'neutral'. A 'positive' response indicates that a patient preferred the 29G/5-bevel needle to the 27G/3-bevel needle; the converse is true for a 'negative' response. A 'neutral' response indicates that the patient did not respond to the question or had no preference for either needle.

#### The two clinical trials

In both trials, analysis of variance (ANOVA) was used to assess pain responses on each pain scale; a 95% confidence interval (CI) was used. The effects included in the ANOVA model were the country, the skin-prick session, the nurse, the volunteer, the injection number and the needle effects. A meta-analysis was conducted to compare the results of the two trials; correlation coefficients between the VB-VAS and the Gracely Box SL pain scale, and between the VAS and VB-VAS were calculated. In the French single-center study, post-hoc pair-wise comparisons were used to rank the different needles according to the level of perceived pain on the VAS and VB-VAS. For both pain scales, a linear model was performed to evaluate the impact of three needle parameters (gauge, bevel geometry and needle-shield material) on pain. The following factors were studied in the model: gauge (27G versus 29G), needle-point geometry (3 versus 5 bevel), needle-shield material (rubber versus TPE), two-way interactions between needle gauge, point geometry, shield material and type of silicone lubricant, volunteer (for VAS evaluated by the subjects) or nurse (for VAS evaluated by the nurses), day (day 1/day 2/day 3), period of day (P1, P2) and order of injection in period (1–4). A second linear analysis was performed in the French single-center study to determine the best candidate needle for pre-attachment to BD Hypak Physiolis™ syringe. The three factors (needle gauge, bevel geometry and needle shield material) were collapsed into one, eight-level factor, which represented the eight test needles. If the eight-level factor 'needle' was statistically significant at risk I error = 0.05, pair-wise comparisons at risk I error = 0.05 were performed using the Tukey multiple comparison method for the p value and confidence limits for the differences of least square means.

## Results

### Clinical Studies

#### Participants

A total of 120 adults with a mean (standard deviation) age of 23.3 (3.8) years were enrolled in the French single-center study and 121 adults aged 35.5 (4.8) years were enrolled in the multi-center study (60 in France and 61 in the USA).

#### Meta-analysis of the correlation between the VAS, VB-VAS and Gracely Box SL pain scale in single- and multi-center studies

There was a strong, positive correlation between the VB-VAS and VAS (r = 0.869; Figure 1) and between the VB-VAS and the Gracely Box SL pain scale (r = 0.836; Figure 2) for volunteer responses. Volunteers had a tendency to use the mid range of the VB-VAS and the extremes of the Gracely Box SL scale. Subjects also had a tendency to use the extremes of the VAS to a greater extent than the extremes of the VB-VAS. ANOVA analysis of the VB-VAS indicated that language (French or English) did not affect the outcome of the scale.

#### Single-center study: Analysis of pain and ease of skin penetration

**Figure 3:**
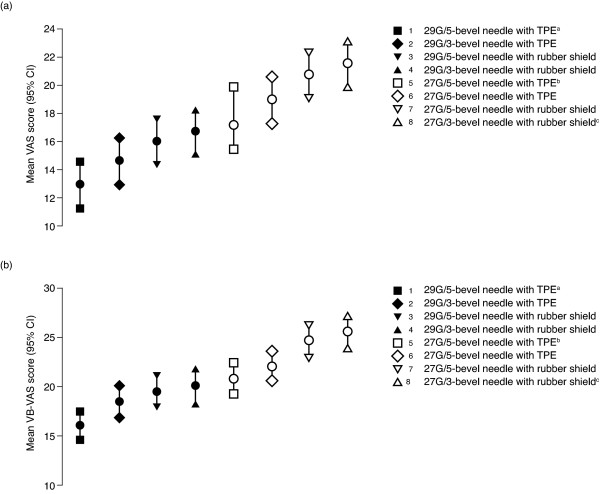
**Volunteers' perception of pain associated with injections using different needle types on (a) the Visual Analog Scale (VAS) and (b) the Verbal VAS (VB-VAS) in the single-center French study (n = 120).** The number next to the key indicates the magnitude of mean pain associated with each needle: 1 = lowest level of pain; 8 = highest level of pain. ^a^Needle currently used for the injection of interferon beta-1a (IFN β-1a), 44 or 22 mcg subcutaneously three times weekly. ^b^Silicone lubricant B was used with this needle; for all other needles, silicone lubricant A was used. ^c^Needle used previously for the injection of IFN β-1a, 44 or 22 mcg sc tiw. CI, confidence interval; G, gauge; TPE, Thermo Plastic Elastomer.

Mean pain scores on the VB-VAS and VAS were lower for all the 29G needles than for the 27G needles, and the two pain scales produced the same ranking of the needles, with the same range of average pain (Figure 3). The needle that is currently used for the injection of IFN β-1a, 44 or 22 mcg sc tiw, the 29G/5-bevel needle with TPE shield, was associated with the least pain; the previous 27G/3-bevel needle with rubber shield was associated with the most pain. The 29G/5-bevel needle with TPE shield was associated with a significantly lower (40% lower) mean pain score on the VAS (12.9; 95% CI: 11.1, 14.6) than the 27G/3-bevel needle with rubber shield (21.5; 95% CI: 19.8, 23.2; p < 0.01). The mean VAS pain score was also significantly lower for the 29G/5-bevel needle with the rubber shield (16.0; 95% CI: 14.3, 17.7) than the 27G/3-bevel needle with rubber shield – a reduction in mean pain score of 26% (p = 0.0002).

Using the VAS, nurses' mean score for skin penetration was significantly lower (70% lower) for the 29G/5-bevel needle with TPE shield (13.5; 95% CI: 11.8, 15.3) than for the 27G/3-bevel needle with rubber shield (43.7; 95% CI: 41.9, 45.5; p < 0.01). The mean VAS score for skin penetration was also lower for the 29G/5-bevel needle with rubber shield (24.0; 95% CI: 22.2, 25.8) than the 27G/3-bevel needle with rubber shield – a reduction in mean pain score of 45% (p < 0.0001).

When the rankings of mean pain scores on the VAS and VB-VAS (Figure 3) are grouped by needle-shield material (rubber or TPE), it appears that volunteers were able to discriminate between needles based on needle-tip geometry – the 5-bevel needles were associated with less pain than needles with 3 bevels. Injections using needles with the TPE shield material were perceived by volunteers as less painful than needles fitted with rubber needle shields, regardless of the needle's outer diameter or the number of gauges. There are insufficient data from this study to establish the influence of lubricant type on individuals' perception of injection-associated pain.

### The five surveys

Overall, 368 patients were enrolled across the five surveys: 11 patients in the Australian pilot survey, 156 in the main Australian survey, 14 in the Danish survey, 109 in the German survey and 78 in the US survey. The questions comparing the pain associated with injections using the 27G/3-bevel and 29G/5-bevel needles were pooled across all five surveys and questions comparing ease of insertion were pooled for the Australian pilot and US surveys. Only the US survey asked unique questions comparing the 27G/3-bevel needle with the 29G/5-bevel needle.

**Figure 4:**
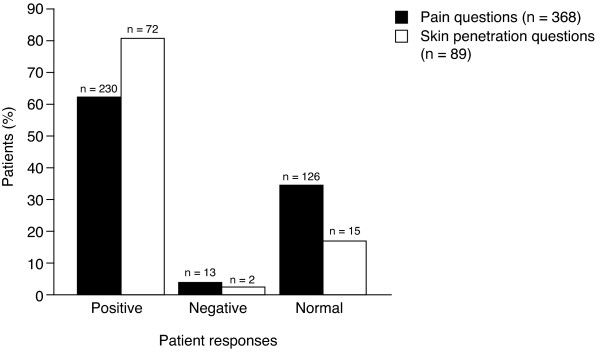
Proportions of patients reporting that the 29-gauge (29G)/5-bevel needle was better (a 'positive' response), similar (a 'neutral' response) or worse (a 'negative' response) than the 27G/3-bevel needle for questions about pain* and skin penetration^† ^(see Table 2** for survey questions).** *Questions about pain were asked in all five surveys. ^†^Questions about ease of skin penetration were asked in the Australian pilot survey (n = 11) and the US survey (n = 78).

Injections were reported to be less painful (positive response) with the 29G/5-bevel needle than with the 27G/3-bevel needle by 63% (230/368) of patients across all five surveys (Figure 4); 34% of patients did not appear to be sensitive to a change in pain (neutral response) and 4% thought the new needle was more painful (negative response). Percentages of positive, negative and normal responses were similar across the Danish, Australian pilot and US surveys; the main Australian survey recorded a slightly lower percentage of positive responses, and the distribution of responses in the German survey was different to the other surveys, with a higher percentage of normal and negative responses. The majority (81%, 72/89) of patients from the Australian pilot and US studies reported that skin penetration was easier with the 29G/5-bevel needle than with the 27G/3-bevel needle, while only two patients (2%) gave a negative response and 15 patients (17%) gave a neutral response (Figure 4). Both surveys recorded similar proportions of positive, negative and normal responses.

Patients from the two Australian surveys were asked whether they noticed a difference between the 29G/5-bevel needle and the 27G/3-bevel needle. Of the 167 responders: 64.7% of patients (n = 108) could differentiate between the two needles (a "yes" response); 18.0% of patients (n = 30) did not notice a difference (a "no" response), and 17.4% of patients (n = 29) gave a neutral response ("not yet indicated" or "not yet tried").

**Figure 5:**
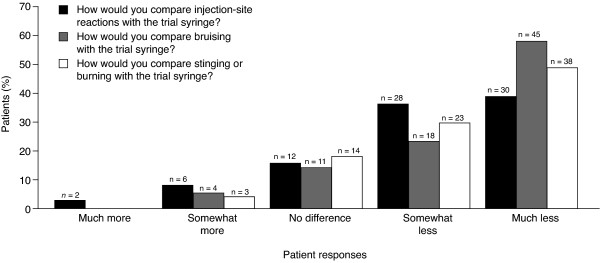
Proportions of patients reporting that the 29-gauge (29G)/5-bevel needle^a ^was better (responses of "much less" or "somewhat less"), similar (a "no difference" response) or worse (responses of "somewhat more" or "much more") than the 27G/3-bevel needle for questions that were unique to the US study (n = 78; see Table 2 for survey questions). ^a^Referred to as the 'trial syringe' by the questionnaire.

For the comparative questions that were unique to the American survey, the majority (74–91%) of responses favored the 29G/5-bevel needle over the 27G/3-bevel needle. Of the 78 patients enrolled in the US study, the majority (74.4%, n = 58) reported a reduction in injection-site reactions with the 29G/5-bevel needle compared with the 27G/3-bevel needle (responses of "somewhat less" or "much less"; Figure 5). The majority of US patients (80.8%, n = 63) also reported less bruising with the 29G/5-bevel needle compared with the 27G/3-bevel needle (responses of "somewhat less" or "much less"; Figure 5). Most patients (78.2%, n = 61) reported less burning and stinging with the 29G/5-bevel needle compared with the 27G/3-bevel needle; responses of "somewhat less" or "much less" (Figure 5). The majority (85.9%, n = 67) of patients reported that the 29G/5-bevel needle was "much better" or "somewhat better" than the 27G/3-bevel needle; 10 patients (12.8%) reported "no difference" and one patient (1.3%) reported that the 29G/5-bevel needle was "somewhat worse". Overall, 91% of patients (n = 71) indicated that they would prefer to use the 29G/5-bevel needle for their regular injections of IFN β-1a, 44 or 22 mcg sc tiw.

## Discussion

The results of the clinical trials in healthy volunteers and surveys in patients with MS indicate that the 29G/5-bevel needle currently used for the injection of IFN β-1a, 44 or 22 mcg sc tiw, is an improvement over the previous 27G/3-bevel needle in terms of pain, ease of insertion and patient satisfaction. Based on the 5040 needle pricks administered in the two clinical trials of healthy volunteers, it appears that not only can individuals discriminate between different needles, based on the gauge, bevel geometry and shield material, but they also thought that the 29G/5-bevel needle was an improvement over the previous 27G/3-bevel needle in terms of subjective pain score on the VAS and VB-VAS. These results are supported by the five surveys in patients with MS who have experience of injections of IFN β-1a, 44 or 22 mcg sc tiw.

When interpreting the results of the clinical trials and the patient surveys, there are a number of precautions that should be noted. The patient surveys are only intended to provide supportive evidence to the more rigorously designed clinical trials. The surveys were unblinded, differed in size (3/5 surveys had fewer than 100 patients), and the results are based on subjective questionnaires, which asked slightly different questions and had different multiple-choice answer options. However, the consistent results between the surveys and the clinical trials support the assertion that the 29G/5-bevel needle is an improvement over the 27G/3-bevel needle, regardless of whether the individuals were healthy volunteers receiving fluid-less injections or patients with MS receiving injections of IFN β-1a. When considering the results of the clinical trials, it should be noted that the VAS and VB-VAS are subjective measures, and the descriptors were modified to accommodate the assessments being made. However, the strong, positive correlation between the VB-VAS and the Gracely Box SL pain scale, which was unmodified, as well as the strong, positive correlation between the VB-VAS and the VAS demonstrate the convergent validity of these pain scales and consistently support the assertion that injections with the 29G/5-bevel needle are associated with less pain than with the 27G/3-bevel needle.

The apparent ability of individuals to discriminate between needles seen in this study is supported by similar findings in a previous study of patients with Type I diabetes and healthy volunteers [1]. Patients with diabetes, who were experienced in carrying out daily sc injections, as well as healthy volunteers, were able to discriminate between good or poor quality needlepoints. This suggests that differences in a needle's gauge and the number of bevels will have a readily noticeable effect on a patient's injection experience. Furthermore, the meta-analysis of the two trials we conducted demonstrate that the VAS, VB-VAS and Gracely Box SL pain scale appear to be reliable tools for assessing individuals' perceived pain associated with injection.

The ranking of the needles on the VAS and VB-VAS scales for pain and ease of insertion indicate that the needle-shield material contributes to an individual's perception of pain associated with injections. The additional analysis we conducted showed that a needle's geometry (gauge and number of bevels) reduced individuals' perceived pain by 40% when comparing the needle currently used to inject IFN β-1a, 44 or 22 mcg sc tiw, with the previous needle. The needle-shield material appeared to account for some of this difference, as demonstrated by the difference in pain scores for the 29G/5-bevel needle with TPE shield and the same needle with the rubber shield versus the 27G/3-bevel needle with rubber shield. The most likely explanation for this difference is that the TPE shield caused less abrasion to the needle during insertion than the rubber shield. The type of silicone lubricant used for injections might have influenced individuals' perceived pain, but further experiments would be required to explore these possibilities.

Although the VAS and Gracely Box SL pain scale were developed and validated for the assessment of pain related to disease conditions and care deliveries, we have successfully managed to discriminate between the performances of different hypodermic needles. We developed the VB-VAS from these two existing pain scales specifically to assess injection-related pain. Indeed, the overall effectiveness of the VB-VAS to detect small differences in noxious skin stimuli, as induced by needle pricks, was the same as the VAS and Gracely Box SL scale. Convergent validity of the three pain scales was confirmed by strong, positive correlations between the VB-VAS and the Gracely Box SL pain scale and the VAS and the VB-VAS. Further evidence of convergent validity of the VB-VAS and VAS was provided by the identical rankings on both scales of the different types of needle, from least painful (29G/5-bevel needle with TPE shield) to the most painful (27G/3-bevel needle with rubber shield). Together, these results indicate that the three pain scales are reliable tools to discriminate needles according to the level of nociceptive stimulation that is elicited.

The findings of the five international surveys in patients with MS support those of the clinical trials of needle pricks in healthy volunteers. A fundamental difference between the surveys and the trials is that the patients with MS in the surveys were injected with IFN β-1a, 44 or 22 mcg sc tiw, and were experienced at self-administration using the previous 27G/3-bevel needle. Importantly, unique questions in the US survey indicated that, in addition to reduced pain, patients reported less bruising, burning and stinging, and fewer injection-site reactions when injecting IFNβ-1a, 44 or 22 mcg sc tiw, using the 29G/5-bevel needle than when using the 27G/3-bevel needle.

To address the potential concern that the thinner 29G/5-bevel needle may be damaged more easily than the 27G/3-bevel needle, a needlepoint drop test was also performed (data on file). After each needle was dropped 90 times from a known height on to a polypropylene sheet, 84% of 29G/5-bevel needles remained free from needlepoint defects compared with only 33% of 27G/3-bevel needles. Thus, despite being sharper and thinner, this engineering study suggests that the 29G/5-bevel needle is less prone to accidental damage during routine use. This is particularly important as injections of IFN β-1a are self-administered by patients with MS.

The efficacy of IFN β-1a, 44 or 22 mcg sc tiw, in the treatment of MS is well established through clinical trials such as the Prevention of Relapses and Disability by Interferon beta-1a Subcutaneously in Multiple Sclerosis (PRISMS) trial and the EVidence of Interferon Dose-response: European North American Comparative Efficacy (EVIDENCE) trial [6-9]. It is expected that the 29G/5-bevel needle will result in additional treatment benefits by improving compliance and patient satisfaction. Of the DMDs that are currently approved for the treatment of patients with relapsing forms of MS, only IFN β-1a, 44 or 22 mcg sc tiw, is supplied to patients in a ready-to-use, pre-filled syringe that is pre-fitted with the 29G/5-bevel needle and TPE shield. In comparison, other formulations of IFN β are administered using thicker needles fitted with rubber shields and/or reconstitution is required before patients can inject.

## Conclusion

The clinical trials in healthy volunteers indicate that injections with the thinner, sharper 29G/5-bevel needle with TPE shield are associated with less pain and greater ease of penetration compared with the previous 27G/3-bevel needle with standard rubber shield. These findings were supported by the results of the surveys, in which patients with MS self-administered IFN β-1a using the 29G/5-bevel and 27G/3-bevel needles. Although needle geometry appeared to be the main factor accounting for reductions in pain, shield material and lubricant may have also contributed to the reduction in pain and should thus be given consideration when designing new needles. It is expected that the apparent improvement in satisfaction with the 29G/5-bevel needle will be manifested by improved compliance in patients with MS who are treated with IFN β-1a sc tiw.

## Competing interests

AJ is a current employee of UCB Pharma SA, Braine l'Alleud, Belgium, and GBB was a previous employee of Merck Serono International S.A. (an affiliate of Merck KGaA, Darmstadt, Germany). LV, WAP, JB and PEL are all employees of BD Medical – Pharmaceutical Systems.

## Authors' contributions

AJ worked as Project Leader. GBB coordinated development activities and logistics, but sadly passed away during the preparation of this manuscript. PEL is BD Medical – Pharmaceutical Systems, Medical Affairs' contributor. LV, WAP and JB led the industrial development and design verification of the 29-gauge needle.

## Pre-publication history

The pre-publication history for this paper can be accessed here:


